# A Selenium-Based Ionic Liquid as a Recyclable Solvent for the Catalyst-Free Synthesis of 3-Selenylindoles

**DOI:** 10.3390/molecules18044081

**Published:** 2013-04-05

**Authors:** Everton G. Zimmermann, Samuel Thurow, Camilo S. Freitas, Samuel R. Mendes, Gelson Perin, Diego Alves, Raquel G. Jacob, Eder J. Lenardão

**Affiliations:** 1 LASOL – CCQFA, Universidade Federal de Pelotas — UFPel, P.O. Box 354, 96010-900, Pelotas, RS, Brazil; 2 GAPAM, Departamento de Química, Universidade do Estado de Santa Catarina, 89219-719, Joinville, SC, Brazil

**Keywords:** selenium ionic liquid, selenium compounds, arylselenylindoles, indoles

## Abstract

The ionic liquid 1-butyl-3-methylimidazolium methylselenite, [bmim][SeO_2_(OCH_3_)], was successfully used as solvent in the catalyst-free preparation of 3-arylselenylindoles by the reaction of indole with ArSeCl at room temperature. The products were obtained selectively in good yields without the need of any additive and the solvent was easily reused for several cycles with good results.

## 1. Introduction

Functionalized indoles, such as 3-arylthioindoles have attracted the attention of researchers in organic synthesis and medicinal chemistry due their potent pharmacological activities, including the inhibition of breast cancer cells [1] and of 5-lipoxygenase, which may increase the antitumor activity of the drug celecoxib [2], and in the treatment of heart disease [3] and HIV [4]. In this sense, there are a range of methods to synthesize this class of compounds, most of them starting from indoles and an electrophilic sulfur reagent [5,6,7,8].

Despite the fact that the usefulness of organoselenium compounds in chemical sciences has already been described in a great number of reviews and books [9,10,11,12,13,14,15] the synthesis of 3-arylselenylindoles and their potential bioactivity have not been extensively studied. These compounds can be obtained by electrophilic cyclization of 2-alkynylanilines with arylselenyl chlorides [16,17] or iodides [18], by the annulation of 2-(*gem*-dibromo(chloro)vinyl)-*N*-methylsulfonylanilines with diselenides [19] or by the cyclization of 2-styrylacetanilides using *N*-phenylselenosuccinimide [20].

The direct selenylation of the easily available indole core, which is a more direct route to 3-arylselenylindoles, was even less explored, as for example in the indoline dehydrogenation using phenylseleninic anhydride, (PhSeO)_2_O [21,22,23] or phenylseleninic acid, PhSeOH [24] (in these works 3-phenylselenylindole was a side product) and in the reaction of 2-acylphenylselenocyanates with phenylhydrazine (only one example) [25]. The reactions between indoles and electropilic selenium species catalyzed by I_2_/FeF_3_ [26] and *p*-TsOH [27] were also described. More recently, Silveira and co-workers [28] used the PhSeSePh/TCCA/MgO system to generate PhSeCl *in situ*, which was reacted with several indoles to give the respective 3-arylselenylindoles in good yields. 

**Figure 1:**
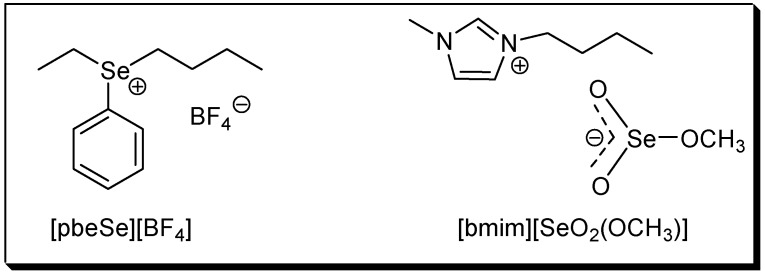
Structures of the selenium-based ionic liquids.

The use of ionic liquids (ILs) as solvent and/or catalyst has attracted much attention in the last years. Because product isolation or catalyst recycling in ILs is very easy and, in some cases, rate accelerations and/or selectivity improvements are also observed, they are regarded as environmentally friendly, green solvents [29,30,31,32,33]. In this context, the use of the new selenium-based ILs phenylbutyl-ethyl selenonium tetrafluoroborate, [pbeSe][BF_4_] and 1-butyl-3-methylimidazolium methylselenite, [bmim][SeO_2_(OCH_3_)] (Figure 1) was recently reported. The selenonium IL was used as an efficient catalyst in several acid-catalyzed reactions [34,35,36], while the selenite IL was employed in the oxidative carbonylation of aniline [37], the base-free oxidation of thiols to disulfides [38] and in the synthesis of vinyl sulfides [39]. 

**Scheme 1:**
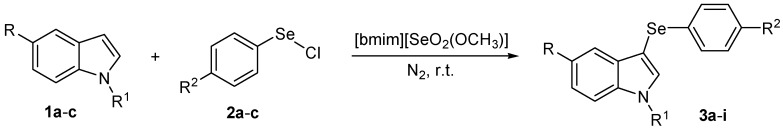
Synthesis of 3-arylselenylindoles using [bimim][SeO_2_(OCH_3_)] as solvent.

To the best of our knowledge, the preparation of 3-arylselenylindoles directly from indoles and electrophilic selenium species under acid-free conditions was not described. In this context, and due our ongoing interest in new applications for selenium-based ionic liquids and selenium-containing compounds, we decide to investigate the use of [bmim][SeO_2_(OCH_3_)] as solvent for the general, catalyst-free arylselenation of indoles to prepare 3-arylselenylindoles (Scheme 1). 

## 2. Results and Discussion

Our initial efforts were made towards the determination of the optimum conditions to perform the reaction. Thus, we chose indole (**1a**) and phenylselenyl chloride (**2a**) to establish the best conditions for the arylselenylation reaction (Table 1).

We examined the effect of temperature, use of nitrogen atmosphere and different ionic liquids, such as [bmin][PF_6_], [bmin][BF_4_], [bmim][SeO_2_(OCH_3_)] and [pbeSe][BF_4_]. Except for [bmim][PF_6_] (Table 1, entry 3), the desired selenylated indole **3a** was isolated in all the tested conditions and the best yields were observed using [bmim][SeO_2_(OCH_3_)] as solvent (78 and 79% yields, entries 1 and 6). In contrast, the selenonium ionic liquid [pbeSe][BF_4_] was not stable under the reaction conditions, affording a lower yield of **3a** among a mixture of diorganyl selenides resulting from the IL (Table 1, entry 2). We also verified the influence of the electrophilic selenium species in the reaction. It was found that when PhSeBr was used the yield decreased to 57% (Table 1, entry 5), while *N*-phenylselenyl phthalimide (PSP) afforded 3-phenylselenylindole **3a** in a similar yield to PhSeCl (79%, entry 6). Since PhSeCl is cheaper than PSP, it is advantageous to use the former reagent instead PSP. It was also observed that when an open atmosphere is used or the mixture is heated, the yield of **3a** decreases. In the second case, decomposition of the IL was observed, with darkening of the solution. Thus, the best reaction conditions were defined as stirring a solution of indole **3a** (1.0 mmol) and phenylselenyl chloride **2a** (1.0 mmol) in [bmim][SeO_2_(OCH_3_)] (1.5 mL) at room temperature under a N_2_ atmosphere for 3 hours.

**Table 1:** Optimization studies for preparation of 3-(phenylselenyl)-1*H*-indole ^a^. 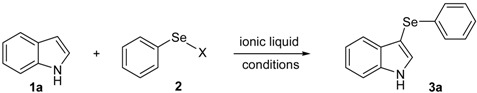

entry	ionic liquid	X	time (h)	yield (%) ^b^
1	[bmim][SeO_2_(OCH_3_)]	Cl	3	78
2	[pbeSe][BF_4_]	Cl	3	39^c^
3	[bmim][PF_6_]	Cl	24	NR^d^
4	[bmim][BF_4_]	Cl	3	28
5	[bmim][SeO_2_(OCH_3_)]	Br	3	57
6	[bmim][SeO_2_(OCH_3_)]	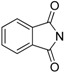	3	79

^a^ The reaction was performed using indole (**1a**, 1.0 mmol) and electrophilic selenium species (1.0 mmol) in ionic liquid (1.5 mL) at room temperature and under a N_2_ atmosphere. ^b^ Isolated yields. ^c^ Decomposition of the IL was observed. ^d^ No reaction.

With these optimized conditions in hands, a detailed study was performed with different indoles and arylselenyl chlorides, showing the generality of the method (Table 2). 

**Table 2:** Synthesis of 3-organylselanylindoles ^a^. 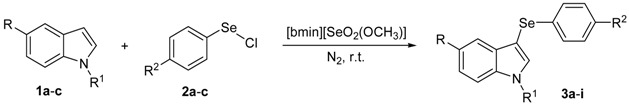

Entry	Indole 1a–c	2a–c	Product 3a–i	Time (h)	Yield (%) ^b^
1	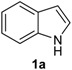	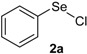	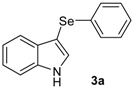	3	78 (74)^c^
2	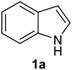	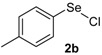	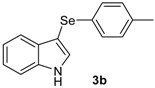	2	73
3	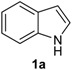	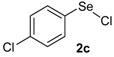	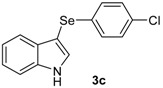	3	55
4	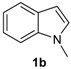	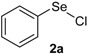	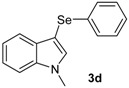	3	68
5	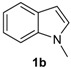	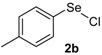	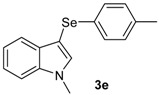	2	65
6	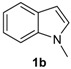	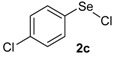	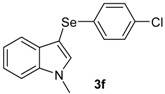	3	53
7	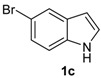	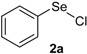	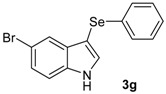	3	74
8	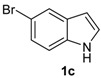	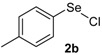	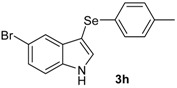	2	65
9	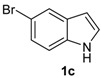	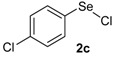	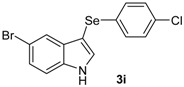	3	62

^a^ The reaction was performed using indole (**1**, 1.0 mmol) and electrophilic selenium species (**2**, 1.0 mmol) in ionic liquid (1.5 mL) at room temperature and under N_2_ atmosphere. ^b^ Isolated yields. ^c^ Reaction performed in a 10 mmol scale and using 6 mL of IL.

From the results listed in Table 2, it can be seen that [bmim][SeO_2_(OCH_3_)] was a good reaction medium to afford a wide range of 3-arylselenylindoles. A possible role of the IL here could be a neutralizing effect in the HCl released in the reaction. 

The presence of electron-donor and electron-withdrawing groups on the selenium species did not affect substantially the yields or the reaction time. Thus, indole (**1a**) reacted with *p*-tolylselenyl chloride (**2b**) under our conditions to afford 3-*p*-tolylselenylindole (**3b**) in 73% yield after 3 h (Table 2, entry 2). Analogously, 4-chlorophenylselenyl chloride (**2c**) afforded the respective 3-arylselenylindole (**3c**) in 55% yield after 3 h after reaction with **1a ** (entry 3). We also extended our protocol to *N*-methyl-1*H*-indole (**1b**), which afforded the 3-arylselenylindoles **3d**–**f** after reaction with **2a**–**c** in slightly lower yields than those obtained for the parent compound **3a** (Table 2, entries 4–6). Similarly, the functionalized 5-bromo-1*H*-indole (**3c**) reacted at room temperature with the arylselenyl chlorides **2a**–**c** in presence of [bmim][SeO_2_(OCH_3_)] to selectively afford the desired 5-bromo-3-(arylselanyl)-1*H*-indoles **3g**–**i** in good yields after short reaction times (Table 2, entries 7–9).

Additionally, a reuse study of the ionic liquid was carried out for the reaction of **1a** with **2a** to obtain **3a**. After stirring at r.t. during 3 h, the reaction mixture was diluted with ether (3 × 5.0 mL). The upper organic phase was washed with water, the solvent evaporated and the product was isolated. The remaining [bmim][SeO_2_(OCH_3_)] was directly reused for further reactions, simple by adding more reagents **1a** and **2a**. It was observed that as the IL was being reused, it gradually darkened, from an initial light yellow to orange and then to red and finally brown, which may be indicative of its decomposition. It was observed that a good level of efficiency was maintained up until the fourth cycle and it dropped in the fifth and sixth cycles (Table 3).

**Table 3:** Reuse of [bmim][SeO_2_(OCH_3_)]. 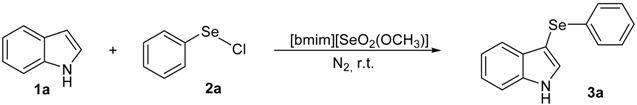

Cycle	Time (h)	Yield of 3a (%) ^a^
1	4	77
2	4	73
3	4	75
4	4	70
5	24	16
6	24	trace

^a^ Yields are given for isolated products.

## 3. Experimental

### 3.1. General

Nuclear magnetic resonance spectra (^1^H- and ^13^C-NMR) were obtained at 200 and 400 MHz on Bruker DPX spectrometers. Spectra were recorded in CDCl_3_ solutions. Chemical shifts are reported in ppm, referenced to tetramethylsilane (TMS) as the external reference. Data are reported as follows: chemical shift (*δ*), multiplicity, coupling constant (*J*) in Hertz and integrated intensity. Carbon-13 nuclear magnetic resonance spectra (^13^C-NMR) were obtained at 50 and 100 MHz on Bruker DPX spectrometers. Spectra were recorded in CDCl_3_ solutions. Chemical shifts are reported in ppm, referenced to the solvent peak of CDCl_3_. Mass spectra (MS) were measured on a Shimadzu GCMS-QP2010 mass spectrometer. Column chromatography was performed using Merck Silica Gel (230–400 mesh). Thin layer chromatography (TLC) was performed using Merck Silica Gel GF_254_, 0.25 mm thickness. For visualization, TLC plates were either placed under ultraviolet light, or stained with iodine vapor, or acidic vanillin. All solvents were used as purchased unless otherwise noted. *p*-Tolylselenyl chloride (**2b**) and 4-chlorophenylselenyl chloride (**2c**) [40] and the ionic liquids [bmim][SeO_2_(CH_3_)] [37] and [pbeSe][BF_4_] [34] were synthesized as described in the literature. 

### 3.2. General Synthesis Procedure

To a mixture of indole **1 ** (1.0 mmol) in [bmim][SeO_2_(OCH_3_)] (1.5 mL) under a N_2_ atmosphere, organylselenyl chloride **2** (1.0 mmol) was added at room temperature and the mixture was stirred for the time indicated in Table 2. The progress of the reaction was monitored by TLC. After the reaction was complete, the product was extracted by successive washings with ether (3 × 5 mL). The upper organic phase was washed with water, dried over MgSO_4_, and concentrated under vacuum. The residue was purified by column chromatography on silica gel using ethyl acetate/hexanes as the eluent. All the compounds were characterized and the corresponding spectral data are listed below:

*3-(Phenylselenyl)-1H-indole* (**3a**) [26]: Yield: 0.213 g (78%). ^1^H-NMR (CDCl_3_, 400 MHz): *δ* = 8.35 (br s, 1H), 7.64 (d, *J* = 7.9, 1H), 7.39–7.43 (m, 2H), 7.22–7.25 (m, 4H), 7.08–7.14 (m, 3H). ^13^C-NMR (100 MHz, CDCl_3_): δ = 136.4, 133.8, 131.2, 129.9, 128.9, 128.7, 125.6, 122.9, 120.8, 120.3, 111.3, 98.2. MS: *m/z* (rel. int.) 273 (6.0), 193 (100.0), 117 (5.3), 77 (21.0).

*3-(p-Tolylselenyl)-1H-indole* (**3b**): Yield: 0.210 g (73%). ^1^H-NMR (CDCl_3_, 200 MHz): *δ* = 8.18 (s, 1H), 7.63 (d, *J* = 7.6, 1H), 7.14–7.32 (m, 7H), 6.91 (d, *J* = 7.6, 1H), 2.20 (s, 3H). ^13^C-NMR (50 MHz, CDCl_3_): δ = 136.5, 135.6, 133.4, 130.8, 130.5, 129.7, 129.4, 122.8, 120.8, 120.4, 111.3, 99.0, 20.8. MS: *m/z* (rel. int.) 287 (2.7), 207 (100.0), 117 (4.9), 77 (14.4).

*3-(4-Chlorophenylselenyl)-1H-indole* (**3c**): Yield: 0.169 g (55%). ^1^H-NMR (CDCl_3_, 200 MHz): *δ* = 8.39 (br s, 1H), 7.59 (d, *J* = 7.7 Hz, 1H), 7.38–7.42 (m, 2H), 7.03-7.29 (m, 6H). ^13^C-NMR (50 MHz, CDCl_3_): δ = 136.6, 132.1, 131.8, 130.3, 129.9, 129.0, 123.2, 121.1, 120.3, 111.4, 98.4. MS: *m/z* (rel. int.) 307 (4.9), 227 (100.0), 116 (12.5), 77 (14.1).

*1-Methyl-3-(phenylselenyl)-1H-indole* (**3d**) [27]: Yield: 0.195 g (68%). ^1^H-NMR (CDCl_3_, 200 MHz): *δ*= 7.63 (d, *J* = 7.8, 1H), 7.07–7.35 (m, 9H), 3.74–3,80 (s, 3H). ^13^C-NMR (50 MHz, CDCl_3_): δ = 137.6, 135.5, 134.2, 130.8, 129.3, 128.9, 125.6, 122.4, 120.5, 120.4, 109.5, 96.4, 33.0. MS: *m/z* (rel. int.) 287 (8.0), 207 (100.0), 130 (18.7), 77 (11.2).

*1-Methyl-3-(p-tolylselenyl)-1H-indole* (**3e**): Yield: 0.196 g (65%). ^1^H-NMR (CDCl_3_, 200 MHz): *δ* = 7,61 (d, *J* = 7.9, 1H), 7.35–6.87 (m, 8H), 3.79 (s, 3H), 2.22 (s, 3H). ^13^C-NMR (50 MHz, CDCl_3_): δ = 137.6, 135.4, 135.2, 130.8, 130.2, 129.7, 129.3, 122.4, 120.6, 120.3, 109.4, 97.0, 32.8, 20.8. MS: *m/z* (rel. int.) 301 (5.4), 221 (100.0), 130 (17.1).

*3-(4-Chlorophenylselenyl)-1-methyl-1H-indole* (**3f**): Yield: 0.170 g (53%). ^1^H-NMR (CDCl_3_, 200 MHz): *δ* = 7.59 (d, *J* = 7.7, 1H), 7.03–7.39 (m, 8H), 3.83 (s, 3H). ^13^C-NMR (50 MHz, CDCl_3_): δ = 137.5, 135.6, 132.5, 131.5, 130.4, 129.9, 128.9, 122.6, 120.5, 120.3, 109.6, 91,7, 33.0. MS: *m/z* (rel. int.) 321 (8.7), 241 (100.0), 130.0 (26.7), 77 (11.5).

*5-Bromo-3-(phenylselenyl)-1H-indole* (**3g**) [26]: Yield: 0.260 g (74%). ^1^H-NMR (CDCl_3_, 400 MHz): *δ*= 8.38 (br s, 1H), 7.74 (s, 1H), 7.38 (d, *J* = 2.2, 1H), 7.09–7.33 (m, 8H). ^13^C-NMR (100 MHz, CDCl_3_): δ = 135.0, 133.3, 132.4, 131.8, 129.0, 128.7, 125.9, 125.8, 122.9, 114.3, 112.9, 97.8. MS: *m/z* (rel. int.) 351 (15.2), 271 (100.0), 192 (73.8), 116 (11.3), 77 (42.3).

*5-Bromo-3-(p-tolylselenyl)-1H-indole* (**3h**) [28]: Yield: 0.237 g (65%). ^1^H-NMR (CDCl_3_, 200 MHz): *δ* = 8.42 (br s, 1H), 7.77 (s, 1H), 7.42 (d, *J* = 2.3, 1H), 7.12–7.30 (m, 4H), 6.95 (d, *J* = 8.0, 2H), 2.36 (s, 3H). ^13^C-NMR (50 MHz, CDCl_3_): δ = 135.8, 135.0, 132.1, 131.8, 129.9, 129.3, 129.2, 125.8, 122.9, 114.3, 112.8, 98.4, 20.9. MS: *m/z* (rel. int.) 365 (18.3), 285 (100.0), 194 (12.7), 91 (42.2).

*5-Bromo-3-(4-chlorophenylselenyl)-1H-indole* (**3i**): Yield: 0.239 g (62%). ^1^H-NMR (CDCl_3_, 400 MHz): *δ* = 8.47 (br s, 1H), 7.71 (d, *J* = 1.7, 1H), 7.41 (d, *J* = 2.5, 1H), 7.25–7.33 (m, 2H), 7.06–7.12 (m, 4H). ^13^C-NMR (100 MHz, CDCl_3_): δ = 135.0, 132.4, 131.8, 131.6, 131.5, 130.0, 129.1, 126.0, 122.7, 114.4, 114.4, 112.9. MS: *m/z* (rel. int.) 385 (5.6), 307 (75.0), 191 (72.1), 115 (46.8), 75 (100.0).

## 4. Conclusions

In summary, we present here the first report on the use of the ionic liquid [bmim][SeO_2_(OCH_3_)] in the selective synthesis of 3-arylselenylindoles. The products were obtained in good yields at room temperature in a relatively short time without the need of any additive. Moreover, the IL could be reused directly for up to four cycles with good performance.
